# Supplementary figures and images for: Assessment of microfilaremia in ‘hotspots’ of four lymphatic filariasis endemic districts of Nepal during post-MDA surveillance
Source: PLoS Negl Trop Dis. 2024 Jan 31;18(1):e0011932. doi: 10.1371/journal.pntd.0011932 (PMC10861036; doi:10.1371/journal.pntd.0011932)

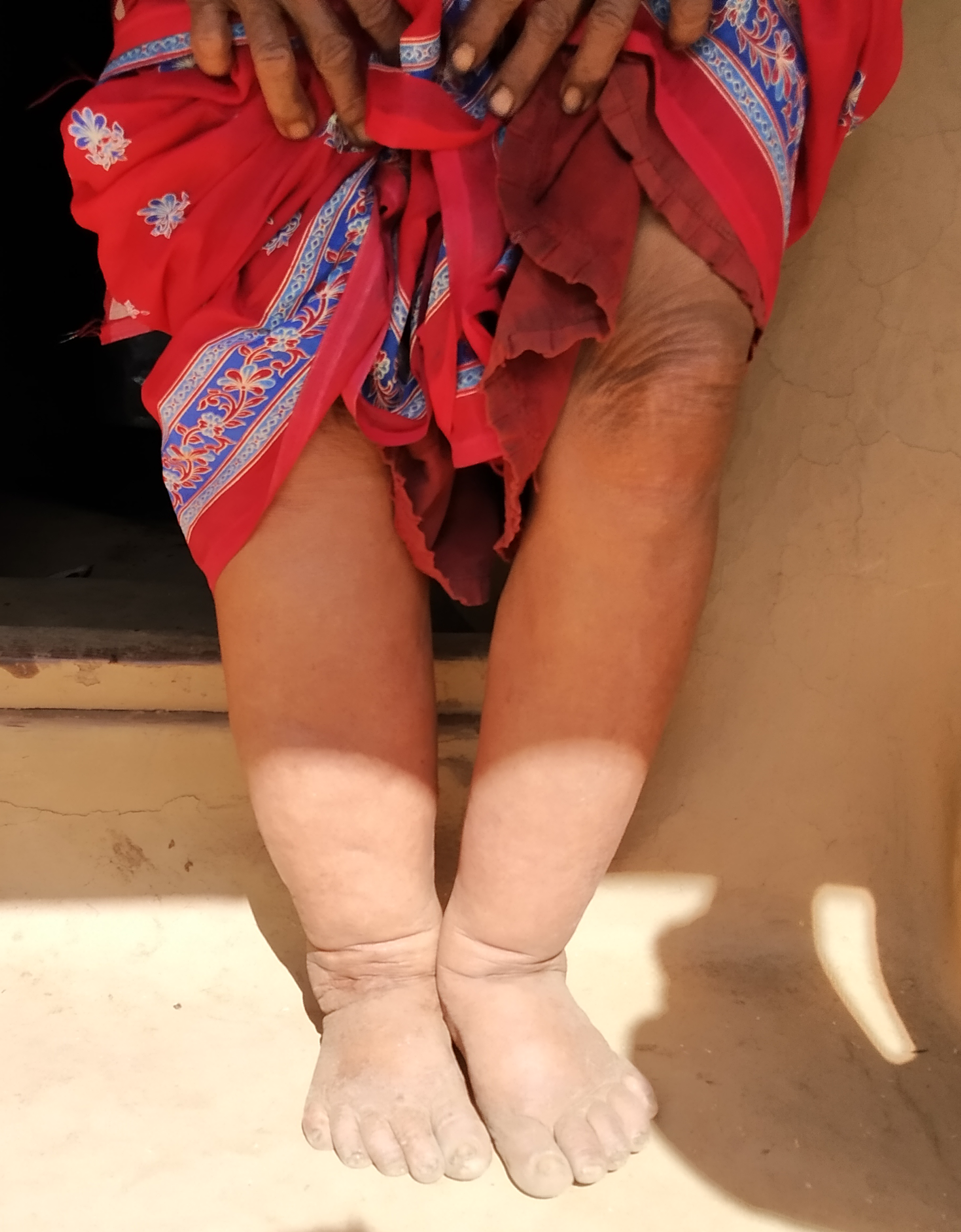

Supplement: S1 Fig — (TIF) [file pntd.0011932.s001.tif]

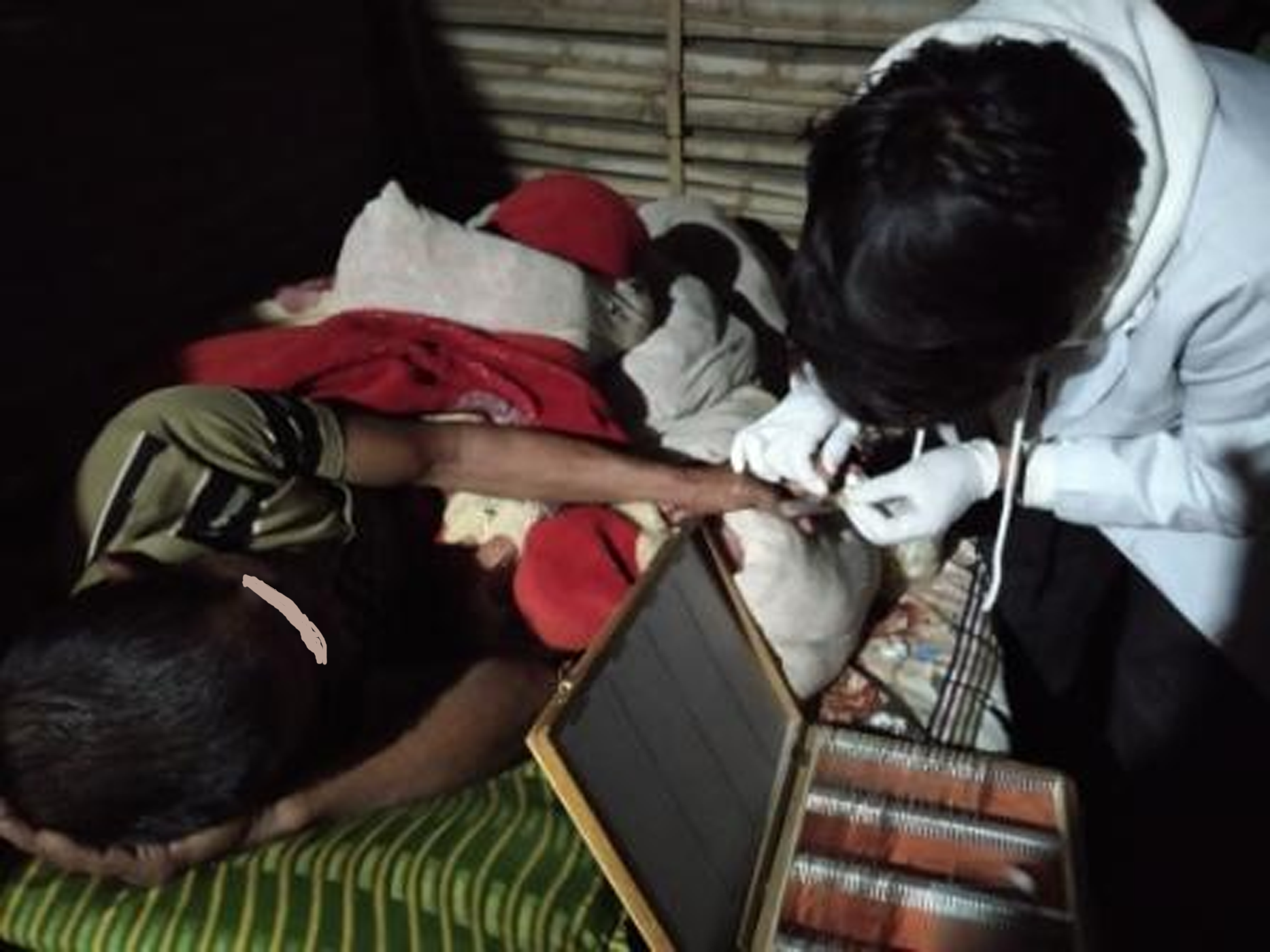

Supplement: S2 Fig — (TIF) [file pntd.0011932.s002.tif]

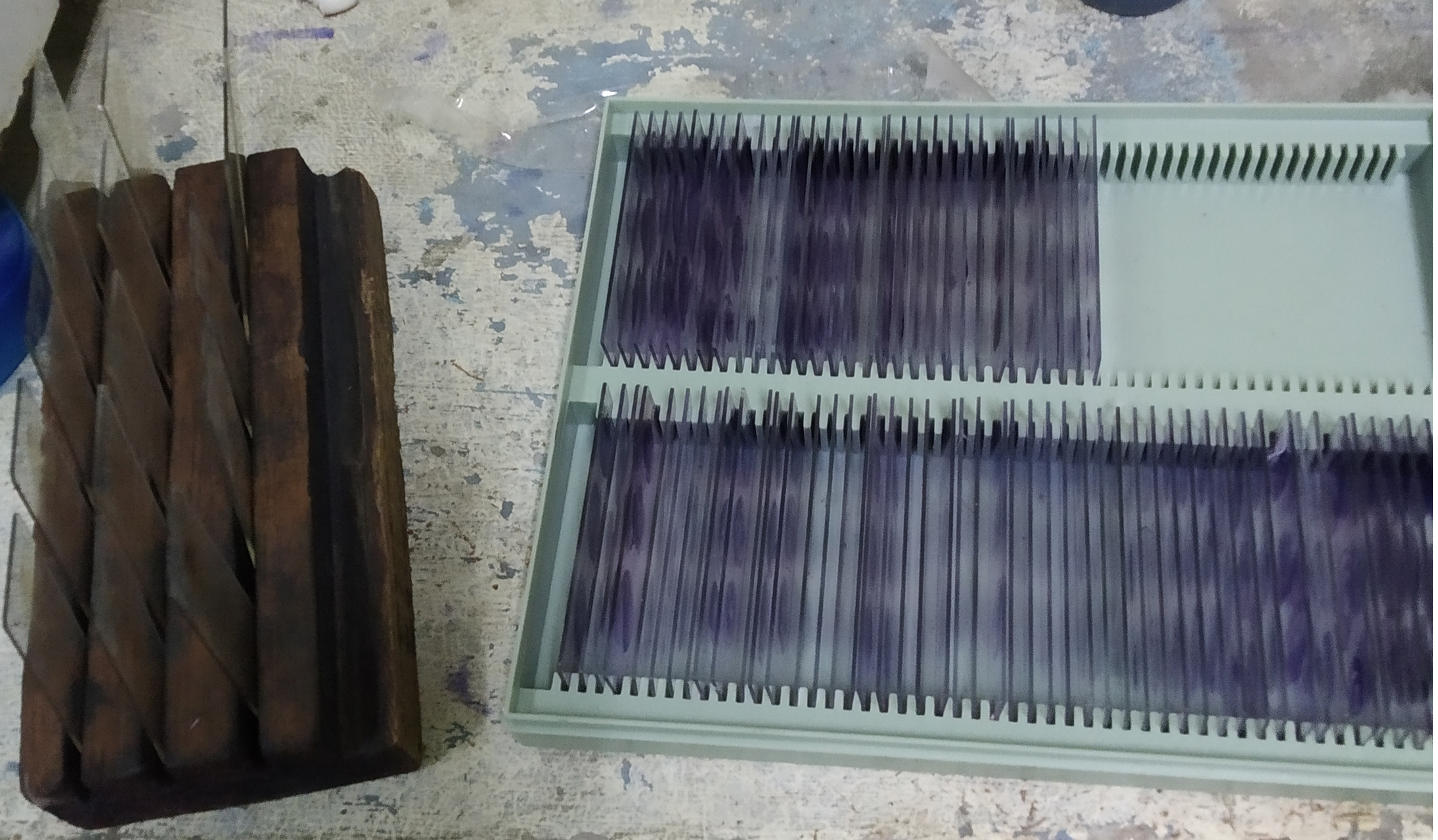

Supplement: S3 Fig — (TIF) [file pntd.0011932.s003.tif]
