# Supplementary material for: Assessment of microfilaremia in ‘hotspots’ of four lymphatic filariasis endemic districts of Nepal during post-MDA surveillance
Source: PLoS Negl Trop Dis. 2024 Jan 31;18(1):e0011932. doi: 10.1371/journal.pntd.0011932 (PMC10861036; doi:10.1371/journal.pntd.0011932)
Supplement: Attachment — Submitted filename: Answer to comments (2).pdf [file pntd.0011932.s004.pdf]

Pramod Kumar Mehta
Central Department of Zoology, Institute of Science and Technology,
Tribhuvan University, Kirtipur, Nepal

Date: 09/21/2023

Dear Dr Jeremiah M. Ngondi, MB.ChB, MPhil, MFPH, Ph.D

Academic Editor

Francesca Tamarozzi

Section Editor

PLOS Neglected Tropical Diseases

"Assessment of microfilaremia in 'hotspots' of four lymphatic filariasis endemic districts of Nepal during post-MDA surveillance" has been revised as per the reviewer's and editor's comments so please consider it for publication.

## Reviewer reports:
## Reviewer #1:

## Methods

-Are the objectives of the study clearly articulated with a clear testable hypothesis stated?

-Is the study design appropriate to address the stated objectives?

-Is the population clearly described and appropriate for the hypothesis being tested?

-Is the sample size sufficient to ensure adequate power to address the hypothesis being tested?

-Were correct statistical analysis used to support conclusions?

-Are there concerns about ethical or regulatory requirements being met?

- The study objective is clearly articulated but the authors may have to consider consistency in the description of the study objective in various sections: Abstract (line 15,16), Author summary (49-51) and Introduction (90-92).

Ans: Corrected

- The study rightly assesses post-treatment lymphatic filariasis (LF)/W. bancrofti infection by nocturnal microfilaremia (mf) (16,17, 125-127).

Ans: Thank you

- The study compares the mf prevalence with LF baseline, pre-TAS and TAS results to make a conclusion of increasing or decreasing infection. However, the study populations of above 9 years (115) is different from the comparison surveys: baseline survey used above 15 years (205-208), pe-TAS is usually uses above 5 years, TAS is usually in 6-7 years. Similarly, all the comparison surveys tested for filarial antigen (206, Table 4) compared to the mf test conducted in the study. The authors indicate that the selection of study age group was based on WHO recommendation (reference 18). However, a review of this reference did confirm this. WHO 2011 LF M&E guidelines age group recommendation for pre-TAS is > 5 years and TAS is 6-7 years.

Ans: Thank you so much for your critical query, we selected the population those were eligible to participate in the MDA program so the present study indicated new/ or past infection with LF. Due to insufficient data on microfilaria before starting of MDA program so we compared antigen prevalence with mf prevalence. The aim of the current study was to correlate school-based antigenemia prevalence of children whose borne after MDA with mf carriers in the community but in revised version we changed. Reference 18 is not correlated so we corrected.

- The authors investigated suspected infection hotspots/persistent transmission sites. Hotspots was defined as foci with "a cluster of antigen-positive cases and mf carriers" after pre-TAS and TAS, but cluster of positive antigen cases or mf cases was not defined. A clear definition of number of antigen or mf positives that constitute a cluster and therefore hotspot to merit further investigation after a district has passed pre-TAS or TAS would 1) allow for reproducibility of the study 2) be helpful for identifying priority sites/clusters/communities for post-treatment or post-validation surveillance.

Ans: Corrected and mentioned in revised version.

- Study site: The authors stated random selection of survey participants from households, it would be important to readers how the households were selected, and what units the households were selected from e.g., community, sub-district, health area etc. This information is not provided.

Ans: Clearly mentioned in revised version.

- The sample size is adequate as it is comparable to site sample size for pre-TAS

Ans: Thank you

- Ethical considerations were adequately covered.

Ans: Thank you

## Reviewer #2:

- The study is a very important one for the global lymphatic filariasis (LF) community as there is little published research on how to detect and respond to areas of ongoing transmission after mass drug administration (MDA) ends. However, the article and the hypotheses statements do not articulate this framing well. For example, it is unclear throughout how the authors define hotspots and whether they are equivalent to districts or sites/villages. The methodology section of the article needs further details, including how sites were selected, how households within sites were selected, and the sampling framework and data source(s). If sites were selected based on pre-TAS or TAS data, it would be useful to include a brief explanation of those surveys in the paper's background section as well. The question of past participation in MDA is becoming more important and a global effort is ongoing to collect this data. As such, it would be helpful to include the exact questions that were asked about past participation in MDA. It would be useful to know what case definitions were used for hydrocele and elephantiasis and the qualifications of those who examined the participants. In terms of ethics, it would be helpful to know what information/treatment was given to participants with clinical conditions.

Ans: Antigen-positive persons were treated with a single dose of diethylcarbamazine (DEC, 6 mg/kg) and albendazole as recommended, while MF-positive cases were treated with a standard dose of DEC (6 mg/kg body wt. for 12 days) (WHO, 2011). We mentioned in revised version. Household, areas and district selection criteria are clearly mentioned in the revised version. Case definition and issues regarding screening of chronic clinical manifestations such as hydrocele and elephantiasis are clearly mentioned in the revised version.

## Reviewer #3:

This is acceptable. But the English needs editing to improve the content and description.

- Should a T-test be used instead of a chi square test?

Ans: Thank you so much. As per our knowledge, we edited the English language in the revised version

## Comments on the results section

-Does the analysis presented match the analysis plan?

-Are the results clearly and completely presented?

-Are the figures (Tables, Images) of sufficient quality for clarity?

### Reviewer #1:

Analysis matches the analysis plan.

- Results are clearly presented

Ans: Thank you

- Figures and tables are of sufficient quality. Titles of tables 2,3 and 5 may be revised to include LF morbidity (lymphedema and hydrocele). Figure 2 may be clearer with a group bar charts for the 4 districts by year.

Ans: Title of tables 2, 3, and 5 corrected. Fig 2 has been changed.

### Reviewer #2:

- The data analysis would be more useful at a site, rather than district level, as disease prevalence and past participation in MDA can vary widely at village level. The analysis of 'trends' is inappropriate and should not be included, given that previous surveys all had different methodologies, diagnostic tests, age groups, etc. these prevalence levels cannot be compared. Instead the current cross-sectional prevalence can be compared to the microfilaremia cut off of 1%. In addition, given the issues with economic migrants in Nepal, it would be helpful to see an analysis of those 25% of individuals who did not enroll in the study, if that data was collected from the households. The methods mention that the study collected data on movement patterns but did not include this data in the results section.
- This is information lacking in the literature about Nepal and critical to the Nepal and India LF programs and should be included.

Ans: Sentinel sites of districts were only selected for the study so the title of the article only represented the sites rather than the districts but somewhere in the text mentioned district representation of this study so we corrected it in the revised version.

**Following are comments on the tables and figures:**

- Figure 2 is not necessary. Would recommend combining the reported coverage as part of a revised Table 4 that includes background information on all districts. This could then be shifted to Table 1 as part of the background of these four districts, not to examine trends but to situate this study in the context of other LF information about the districts. This table could include population; baseline prevalence, diagnostic test and year; pre-TAS 'prevalence' diagnostic test and year; TAS diagnostic test, year, critical cut off and number of positives; and reported MDA coverage by year.

Ans: Changed in the revised manuscript.

- Current Table 1 would be more useful with rows for each site, and columns for 'eligible' and sampled populations, disaggregated by sex and age group. I would consider having a table with demographic characteristics and a separate table(s) with X2 and p-values.

Ans: Changed in the revised version.

- Current Table 5 would be helpful to include results by site, as well as breakdown of those treated in last MDA and ever treated by age group and sex as well. Past participation results are usually analyzed by asking about participation in the most recent MDA and in all MDAs, including the most recent. If this was also how the authors analyzed it, their results of 75% in most recent and 72% ever participated do not make sense.

Ans: Individuals who participated in previous any rounds of MDA included in reported drug uptake in previous MDAs i.e. 72% (we tried to ask the participant for the total number of rounds of MDA participated but most of the individuals were not memorized past all events so in this group of individuals may be participated in one/ or all or 2, 3,….. so on except last rounds). We just asked the individuals for participation in the last rounds of MDA to be included in reported drug uptake in the last MDA rounds i.e. 75% ( All these groups of individuals last rounds of MDA and may all/ or 1, 2….so on). For minimization of recall bias we just asked the participation in the last and previous rounds of MDA)

## Reviewer #3: -

- When describing the mf prevalence, it will be useful to provide the percentage per district in the text, in addition to the Table that was provided.

Ans: Changed in revised manuscript.

-It would be useful to break the age groupings further to assess whether there were infections in children less than 9 years, and also to show the infection levels in different age categories. e.g. <10, 10-20, 21-30, 31-40, etc... The findings should also be discussed appropriately.

Ans:

- Check Tables 3 and 4. The mf prevalence in Mahottari is indicated as 4.4 in one Table and 5.4 in the other Table.

Ans: Corrected

- Table 4. *, symbols indicate antigen prevalence above the critical value. there was no mention of assessing antigen prevalence in the methods or results. This is not clear and should be clarified, as it leaves me confused.

Ans: * symbols indicate MF prevalence so corrected

## Conclusions

-Are the conclusions supported by the data presented?

-Are the limitations of analysis clearly described?

-Do the authors discuss how these data can be helpful to advance our understanding of the topic under study?

-Is public health relevance addressed?

## Reviewer #1:

- The mf prevalence recorded in two of the study sites surveyed in Dhading (5.8%) and Mahottari (5.4) would be concerning to the national program as they are above the expected threshold > 1%. This is an important finding. However, since the survey focused on suspected high prevalence/"hotspots" sites (Figure 1) in districts in the post-treatment surveillance phase the authors may limit conclusions to the survey sites/communities rather than the districts (175,176, 231-233).

Ans: Corrected

- The authors observe increasing trend of mf in two districts and otherwise in two other districts (181-184, Table 4). However, this is unsupported by the data presented 1) only one data mf prevalence data point is presented out of the 4 data points 2) data in Table 4 does show increasing mf or antigen prevalence trend for any of the four districts.

Ans: Baseline prevalence and LF antigen prevalence of TAS reports are kept in the background of the study whereas the current Mf prevalence concluded based on the critical cut of value in the revised version of the manuscript.

- Line 53-55: Authors may need to show how the study findings support recommendation for Xenomonitoring and antigen testing.

Ans: Increased MF carriers in the current study in community people may spread the infection to individuals through vector mosquitoes so those who never participated in MDA rounds could be checked for an antigen which is a proxy indicator of new infection and infection/ or infectivity in vector mosquitoes indicates the proofing of local transmission. All these indicators could be useful for further intervention in the study areas so our results support a recommendation for xenomonitoring and antigen testing in community people.

- The limitation of the study and analysis were not presented.

Ans: Mentioned in the revised version of the manuscript.

## Reviewer #2:

- As mentioned above, analysis of trends is not appropriate to do. Instead, the analysis, discussions and conclusion should focus on who is infected, how that links to past MDA participation, and recommendations on how the national program should respond. In addition, it would be useful to the global community to know the authors' recommendations on how to find 'hotspots', how to investigate them and how to respond in a programmatic context. No limitation section is included. It would be worthwhile to add this to the discussion.

Ans: Thank you so much for your constructive comments. Analysis of trends has been changed and we linked to past participation in MDAs round and based on this linkage we concluded along with discussed in the revised manuscript.

## Reviewer #3:

- No conclusions were provided in the discussion

Ans: Conclusions are mentioned in the discussion in the revised manuscript.

## Editorial and Data Presentation Modifications?

Use this section for editorial suggestions as well as relatively minor modifications of existing data that would enhance clarity. If the only modifications needed are minor and/or editorial, you may wish to recommend "Minor Revision" or "Accept".

## Reviewer #1:

Authors to look at consistency in use of LF infection, LF cases

- Effective MDA treatment coverage: ≥65% of total population.

Ans: Corrected

- Duration of last MDA and study may affect recall of question on participation in last MDA (187-189). Not clear time of study and last MDA.

Ans: Recall bias mentioned in the limitation of the study. Time of study and last MDA round are mentioned in the revised manuscript.

- Reconcile when LF program, MDA started, MDA stopped- 2003/2001, 2017, 2018

Ans; Clearly mentioned in the revised manuscript.

- Significance of mf prevalence by sex, age (≥ 41 years); lymphedema by age group: review interpretation of results (169,170) and Table 2 (p-values)

Ans: Mentioned and revised

## Reviewer #2:

- This article could benefit from an editor. Given that and the other major revisions stated above, it is not worthwhile to include all the minor modifications here that are needed.

Ans: Revised

## Reviewer #3:

- Significant language editing is required to make the paper acceptable for publication.

Ans: We revised but if you feel again to revise please let us know.

## Summary and General Comments

Use this section to provide overall comments, discuss strengths/weaknesses of the study, novelty, significance, general execution and scholarship. You may also include additional comments for the author, including concerns about dual publication, research ethics, or publication ethics. If requesting major revision, please articulate the new experiments that are needed.

### Reviewer #1:

- The study is important for efforts to leave no foci of infection behind as countries make progress towards elimination of LF as a public health problem. The study is also important as a guide for country programs to target sites for post-treatment and post-validation surveillance. A clear definition of what constitutes a cluster of positive cases of concern to merit further investigation for persistent infection as in this study is fundamental to the manuscript. The authors also need to ensure that all conclusions are supported by the study. Some level of editing for consistency would enhance understanding of readers and flow.

Ans: we clearly mentioned the criteria for the selection of study areas and made a conclusion based on strong study support in the revised manuscript.

### Reviewer #2:

- The study represents an important entry into the global community about how to detect and respond to areas of potential ongoing transmission after MDA is stopped. However, more details are needed about methodology to understand how they detected these areas, and how they sampled the population. The analysis of the results by comparing with previous surveys is inappropriate. More analysis could be done about who was infected (looking at age, sex, occupation, movement patterns) and how past participation linked to infection status. It would be useful to include a discussion of how to respond to these areas and how the Nepal and other programs might translate this research into programmatic activities. With these major revisions, the study would be significant to the LF community as many programs move into post-MDA surveillance.

Ans: Revised

## Reviewer #3:

The authors assessed the mf prevalence in hotspot districts in Nepal, after the cessation of MDA. The findings of the study are interesting and the paper is of importance to the field and sustaining the LF elimination achievements. However, there are a number of issues that need to be addressed before it can be acceptable for publication.

- A strong language editing is recommended

Ans: As per our knowledge we revised

- Kindly provide a more accurate description of the timelines in the abstract and main text. In the abstract, if MDA was started in 2001, that gives 15 rounds of MDA by 2016 and not 6 rounds of MDA. In the methods there is mention of 6 to 11 rounds of MDA from 2007 to 2017. These two different description of the MDA timelines are conflicting.

Ans: Revised and clearly mentioned

- Please present the mf data in the abstract.

Ans: Revised

- Kindly discuss the impact of recall bias on the assessment of MDA participation.

Ans: Discussed in revised

- Under the discussion, a concluding paragraph of the study will be useful.

Ans: Mentioned in revised

- Given the results, kindly discuss the limitations of TAS in the decision to stop MDA and what can be done to improve the TAS processes.

Ans: Mentioned in revised
