# Supplementary material for: Assessment of microfilaremia in ‘hotspots’ of four lymphatic filariasis endemic districts of Nepal during post-MDA surveillance
Source: PLoS Negl Trop Dis. 2024 Jan 31;18(1):e0011932. doi: 10.1371/journal.pntd.0011932 (PMC10861036; doi:10.1371/journal.pntd.0011932)
Supplement: Attachment — Submitted filename: Answer to comments.pdf [file pntd.0011932.s005.pdf]

Pramod Kumar Mehta
Central Department of Zoology, Institute of Science and Technology,
Tribhuvan University, Kirtipur, Nepal
Date: 12/26/2023

Dear Dr Jeremiah M. Ngondi, MB.ChB, MPhil, MFPH, Ph.D
Academic Editor
Francesca Tamarozzi
Section Editor
PLOS Neglected Tropical Diseases

"Assessment of microfilaremia in ‘hotspots’ of four lymphatic filariasis endemic districts of Nepal during post-MDA surveillance" has been revised as per the reviewer's and editor's comments so please consider it for publication. Apart from this revision if you feel further revision please let us give opportunities for needy correction. Thank you so much for your constructive comments. We got a lot of learning opportunity.

## Reviewer's Responses to Questions

**Methods:**

-Are the objectives of the study clearly articulated with a clear testable hypothesis stated?

-Is the study design appropriate to address the stated objectives?

-Is the population clearly described and appropriate for the hypothesis being tested?

-Is the sample size sufficient to ensure adequate power to address the hypothesis being tested?

-Were correct statistical analysis used to support conclusions?

-Are there concerns about ethical or regulatory requirements being met?

### Reviewer #1: (No Response)

### Reviewer #2: -

- The definition of hotspot is still slightly unclear. Lines 16-17 note that the TAS in sentinel sites was below threshold. Usually, sentinel sites are sampled in pre-transmission assessment surveys (pre-TAS), while TAS randomly samples ~30 clusters (schools or villages) in a district. The number of positive children in a TAS are compared to a critical cut off (roughly equal to 2% antigenemia) to see if MDA can be stopped. My guess is that TAS in these 4 districts passed, e.g. was less than the critical cut off, but certain clusters had multiple positive cases and these were classified as hot spots and erroneously called sentinel sites. However, this needs to be more explicitly defined in the paper and the abstract, specifically: was pre-TAS or TAS data used to determine hotspots? what constituted multiple positive cases, 2, 3, 4?

Ans: Thank you so much for the critical questions and valuable suggestions which could be made our article to be constructive. Defined hotspots clearly mention in revised version of manuscript. In our country, school-based TAS survey (Cluster) was carried out so based on TAS survey we considered the hotspots. TAS in all these 4 study districts were passed. Number of antigen-positive cases in hotspots of selected districts were mentioned in revised version. Critical cut off value during TAS mentioned in cluster so due to lack of individual district critical cut off value we are unable to mention number of positive cases vs critical cut off value. Please consider it.

- Lines 118-120 The number of positives found in each cluster should be noted

**Ans:** Mentioned in revised manuscript.

- In the introduction, a short summary of TAS methodology would be appropriate to include around line 90-91.

Ans: Included.

- The specific sampling methods still requires more editing to be understandable. I would recommend discussing sample size and number of households at site level -currently it is

discussed in totals, at district and site level and is difficult to follow. Table 2 helps capture some of this at district and total levels. However, if the sample size and the results are truly at the site level, the other tables should also be constructed to show site-level results.

Ans: Corrected in revised manuscript.

- Line 135 Methods might flow better to start with required sample size and then discuss the number of households needed to be selected to meet this sample size.

Ans: Corrected

- Line 139 I am not a sampling expert, but expecting 50% LF prevalence in a site (when known prevalence was nearer to 3-5%) seems to be inappropriate.

Ans: Due to lack of MF prevalence data before starting of the study, we calculated the sample size based on probability of prevalence i.e.50% probability of prevalence of LF so by using this formula to calculate the sample size seems to be appropriate for this study.

Lines 185-189 Please include at which levels (site, district, total) these analyses were conducted.

Ans: Mentioned in revised manuscript

Table 3 - results should be presented by site, not total

Ans: Corrected

**Reviewer #3:** These are accpetable.

## <u>Results</u>

-Does the analysis presented match the analysis plan?

-Are the results clearly and completely presented?

-Are the figures (Tables, Images) of sufficient quality for clarity?

**Reviewer #1: (No Response)**

**Reviewer #2:**

- The results are presented by total, by district and sometimes by hilly v Terai. As in the methods section, some clarity should be given in presenting at site level, by district and total. Perhaps a separate section and table could look at hilly v Terai if that is critical to the paper. (I'm not sure what the comparison of two districts within the Terai and two within the hilly region as in Table 3 tells us, other than districts/sites are very different.)

Ans: we compared the data within hotspots of 2 hilly districts and similarly within the hotspots of Terai 2 districts but not compared between the hilly and Terai regions. For geographical separation, we used the regions.

Table 3 - Consider bolding results which are significant so that the reader can easily see which ones were significant.

Ans: Corrected

Lines 205-207 Phrasing such as 'much improvement' implies comparison with previous time points. Since that is not able to do be done due to previous different survey methodologies, rephrase to simply 'Mf prevalence is lower than the 1% cut off WHO recommends to stop MDA.'

Ans: Corrected

Line 236 - 'MDA coverage' usually refers to community coverage. In this instance, I believe the 65% is referring to '65% of the study participants noted they participated in the last MDA.'

- Would recommend including a table that reports by site the percentage of participants who had never participated in any MDA and the percentage Mf positive of those who had never participated in any MDA. To answer this, I think the researchers will have to use only those participants who said no to participating in the last MDA round and those who also said no to participating in any other previous rounds.

Ans: Corrected

## Reviewer #3: These are acceptable

## Conclusions

-Are the conclusions supported by the data presented?

-Are the limitations of analysis clearly described?

-Do the authors discuss how these data can be helpful to advance our understanding of the topic under study?

-Is public health relevance addressed?

## Reviewer #1: (No Response)

## Reviewer #2: -

- One complicating factor to this analysis is that Mahottari failed TAS3 in 2019, Bara failed TAS2 in 2017, and Laltipur was split into rural and urban areas for TAS. It's unclear when the data for this study were collected, but given that the article will be published in 2023/2024, they should address how their data links to the above survey results. E.g., line 116 notes that MDA has been stopped, which is not true at present.

Ans: This study was designed in 2018 and based on the TAS1 report (Unpublished TAS report of EDCD), all 4 districts had passed the TAS1 which we showed in Table 1 so we selected these 4 study areas. We collected the data from Lalitpur in 2019, Bara in 2022 and Lalitpur in 2022. During the collection of data, all these districts were stopped the MDA. Lalitpur districts are split into urban and rural and clusters of antigen-positive cases were found in rural areas so we collected the data from rural areas of Lalitpur. In Mahottari TAS3 failed in 2019 which data was not published and EDCD till 2022, further rounds of MDA were not implemented but after this study further rounds of MDA started in 2023 so during our study MDA had been stopped in Mahottari. Based on TAS1 report of Bara we designed current study, TAS 2 failed

in Bara in 2017 and further 4 rounds of MDA was completed before starting of current study which is mentioned in article.

- Line 270-271 is unclear to me - please revise.

Ans: Revised.

- Lines 279-281 I believe some of the rounds in Lalitpur did not reach effective coverage (e.g. 65%) so did not count to the 5 rounds needed by WHO in order to move to pre-TAS and then TAS to stop MDA. In addition, please confirm the data on a number of rounds before TAS1 - this does not match with other data.

Ans: In Lalitpur, MDA was started in 2010 and six rounds of MDA were reached in 2015 but in between some MDA rounds did not reach 65% so extended up to 8 MDA rounds and the program was stopped after 2017. Current study, we could not be considered the coverage just we compared the current prevalence to a number of MDA rounds. In some of the districts seems to have not followed the exact WHO TAS guideline. Some of the data related to MDAs are not getting properly so we are getting problems in exact analysis. Please consider it.

- Lines 281-284 Note that WHO has not found evidence that biannual treatment is more effective than annual treatment (see Guidelines for LF treatment). Instead, I wonder if reported coverage was accurate or if there were groups of people not participating who continued to harbor microfilaremia. In addition, are you recommending that if a hotspot is found after TAS passes, that the entire district should restart MDA? Or are you recommending focal treatment be done in that hotspot only?

Ans: WHO has not found evidence that biannual treatment is more effective than annual treatment but in some research article supported the biannual treatment strategy which is supported by reference no 24. We think as a researcher each and every evidence of WHO only should be

supported to our data so sometimes others researcher data could be followed. In our study MF is not harbor only in group of individuals who never been participated in MDA. Our data only supported for focal treatment only. Mentioned in revised version of manuscript.

- Line 321 - I would not link baseline prevalence in Nepal to number of rounds needed, given issues with baseline prevalence data collection in Nepal, as well as varying reported and surveyed coverage in individual MDA rounds. Your study wasn't designed to study that association.

Ans: As per our knowledge we compared the current data with baseline prevalence, number of MDA rounds and coverage. MDA coverage data and baseline prevalence data were collected by respective departments and we have already mentioned in limitations of the study so please consider this association.

## Reviewer #3: These are acceptable.

### Editorial and Data Presentation Modifications?

Use this section for editorial suggestions as well as relatively minor modifications of existing data that would enhance clarity. If the only modifications needed are minor and/or editorial, you may wish to recommend “Minor Revision” or “Accept”.

## Reviewer #1: (No Response)

**Reviewer #2:** Line 49 - spell out Wuchereria

Line 53 - delete 'in sentinel sites'

Ans: Deleted

Line 54 - not sure what is meant by 'residual' - presumably these were the first time the participants were tested for antigen. Delete.

Ans: Deleted

Line 57 - No evidence this is resurgence of infection if we don't have comparable data. instead 'prevalence above threshold'

Ans: Changed

Line 83 - CFA prevalence during TAS hasn't been used in modeling to determine number of rounds of MDA needed

Ans: Deleted

Lines 113-118 and Table 1 - TAS results should be presented as number positive vs a critical cut off and not as a percentage positive, as due to survey design creating a prevalence from the survey needs a special analysis

Ans: TAS was carried out in cluster-based sampling methods and critical cut off values were fixed based on cluster of districts so due to lack of individual district critical cut off value we are unable write a number of positive vs critical cut-off..

Table 1 - Please confirm - I believe baseline prevalence was Mf (and not antigen) in Bara, lalitpur, and Mahottari.

Ans: We ensure during follow up study only MF survey was done in Bara, Lalitpur and Mahottari but during baseline survey, antigen test was carried out. Due to lack of MF survey data we unable to compare to current study.

Line 289 - The use of 'resurgence' is likely not appropriate since we don't have Mf data from a similar age group from those sites.

Ans: Changed

## Reviewer #3: (No Response)

**Summary and General Comments**

Use  this section to provide overall comments, discuss strengths/weaknesses of the study, novelty, significance, general execution and scholarship. You may also include additional comments for the author, including concerns about dual publication, research ethics, or publication ethics. If requesting major revision, please articulate the new experiments that are needed.

**Reviewer #1:** Comments on the first draft have been adequately addressed. The readability of the manuscript has been improved. I

**Reviewer #2**: In general, the article includes very interesting data but more work is still needed to articulate the definition of hotspots, how the sampling was done, and present the results by site/district/total.

Recommending to do night blood sampling of adults in TAS (cluster based surveys of >1000 people) is likely a recommendation very difficult for most programs to implement. Are there recommendations that could address how to identify and follow up potential hotspots? Do you think it's feasible to do community night blood surveys of ~500 people in hotspots instead?  And what would you recommend the program do for follow up, beyond just treating those who were found positive?

Ans: Night blood surveys of ~500 people and focal treatment of all individuals in hotspots is recommended

**Reviewer #3:** This is a much improved version of the manuscript. My comments have been addressed. Some minor grammatical errors and language editing is still required. However, I believe these will be covered during the proof stage before publication.

**Figure Files:**

While revising your submission, please upload your figure files to the Preflight Analysis and Conversion Engine (PACE) digital diagnostic tool, https://pacev2.apexcovantage.com. PACE helps ensure that figures meet PLOS requirements. To use PACE, you must first register as a user. Then, login and navigate to the UPLOAD tab, where you will find detailed instructions on how to use the tool. If you encounter any issues or have any questions when using PACE, please email us at figures@plos.org.

Ans: All figures are corrected in PACE

## Data Requirements:

Please note that, as a condition of publication, PLOS' data policy requires that you make available all data used to draw the conclusions outlined in your manuscript. Data must be deposited in an appropriate repository, included within the body of the manuscript, or uploaded as supporting information. This includes all numerical values that were used to generate graphs, histograms etc.. For an example see here: http://www.plosbiology.org/article/info%3Adoi%2F10.1371%2Fjournal.pbio.1001908#s5.

## Reproducibility:

To enhance the reproducibility of your results, we recommend that you deposit your laboratory protocols in protocols.io, where a protocol can be assigned its own identifier (DOI) such that it can be cited independently in the future. Additionally, PLOS ONE offers an option to publish peer-reviewed clinical study protocols. Read more information on sharing protocols at https://plos.org/protocols?utm_medium=editorial-email&utm_source=authorletters&utm_campaign=protocols

## References

Please review your reference list to ensure that it is complete and correct. If you have cited papers that have been retracted, please include the rationale for doing so in the manuscript text, or remove these references and replace them with relevant current references. Any changes to the reference list should be mentioned in the rebuttal letter that accompanies your revised manuscript. If you need to cite a retracted article, indicate the article's retracted status in the References list and also include a citation and full reference for the retraction notice.

In compliance with data protection regulations, you may request that we remove your personal registration details at any time. (Use the following URL: https://www.editorialmanager.com/pntd/login.asp?a=r). Please contact the publication office if you have any questions.

Ans: Re-checked but if you feel correction please let us know.
